# A low-cost wind tunnel for bird flight experiments

**DOI:** 10.1007/s10336-021-01945-2

**Published:** 2022-01-19

**Authors:** Herwig A. Grogger, Martin Gossar, Michael Makovec, Johannes Fritz, Katharina Neugebauer, Frederik Amann, Bernhard Voelkl

**Affiliations:** 1 Engineering Department, University of Applied Sciences Joanneum, Alte Poststrasse 149, 8020 Graz, Austria; 2 Waldrappteam, 6162 Mutters, Austria; 3 Animal Welfare Division VPHI, University of Bern, Bern, Switzerland

**Keywords:** Wind tunnel, Bird flight experiments, Northern bald ibis

## Abstract

A blower-type wind tunnel for physiological bird flight experiments has been developed, constructed and evaluated. Since the birds to be investigated are rather big (Northern Bald Ibis, *Geronticus eremita*), the cross-sectional area of the test section measures 2.5 m × 1.5 m. The maximum achievable flow speed is approximately 16 ms^−1^. The wind tunnel exhibits a flexible outlet nozzle to provide up- and downdraft to allow for gliding and climbing flights. The current paper describes in detail the layout, design and construction of the wind tunnel including its control. Numerical simulations of the flow and measurements of the velocity distribution in the test section are presented. Apart from a non-homogeneous flow region in the mixing layer at the boundaries of the free jet, the test section exhibits a very even velocity distribution; the local speed deviates by less than two percent from the mean velocity. The turbulence intensity inside the test section was measured to be between 1 and 2%. As a constraint, a limited budget was available for the project. Four northern bald ibises were hand-raised and trained to fly in the wind tunnel.

**Supplementary Information:**

The online version contains supplementary material available at 10.1007/s10336-021-01945-2.

## Abstract (de)

**Ein kostengünstiger Windkanal für Vogelflugexperimente**

Ein kostengünstiger Windkanal für Vogelflugexperimente wurde entwickelt, gebaut und vermessen. Nachdem die zu untersuchenden Vögel relativ groß sind (Waldrappe, *Geronticus eremita*), misst der Auslassquerschnitt des Windkanals 2,5 m × 1,5 m. Die darstellbare Maximalgeschwindigkeit beträgt etwa 16 ms^−1^. Der Windkanal weist eine vertikal verstellbare Auslassdüse auf, um auf- und abwärtsgerichtete Strömungen realisieren zu können, womit Gleit- und Steigflüge simuliert werden. Der vorliegende Artikel beschreibt detailliert die Auslegung, die Konstruktion und den Bau des Windkanals und seiner Steuerung unter der Randbedingung eines sehr beschränkten Budgets. Eine numerische Simulation der Strömungsverhältnisse und Messungen der Geschwindigkeitsverteilung im Testbereich werden präsentiert. Abgesehen von inhomogenen Strömungsbereichen in der Mischungszone des Freistrahls weist die Messstrecke eine sehr gleichmäßige Geschwindigkeitsverteilung auf: die lokale Geschwindigkeit weicht um weniger als 2% von der mittleren Geschwindigkeit ab. Weiters wurde die Turbulenzintensität in der Messstrecke gemessen. Sie beträgt zwischen 1 und 2%. Vier Waldrappe wurden handaufgezogen und darauf trainiert, im beschriebenen Windkanal zu fliegen.

## Introduction

Scientific experiments with flying birds can be performed when the birds are moving in their natural environment, namely in free air. The birds are equipped with instruments to collect or send the desired data, though the additional load negatively influences the bird’s flight performance due to the added mass and the changed aerodynamics (Obrecht et al. 1988; Schmidt-Wellenburg et al. 2008; Pennycuick et al. 2012). The increased drag and the disturbed flow over its body increase the bird’s required power to fly; it has been estimated that the added weight and increased drag of even small devices can decrease the flight range of small migrants significantly (Bowlin et al. 2010). Furthermore, the mounting of the instrument housing on the bird’s body may hinder its movements and could involve other risks (Bowlin et al. 2010; Fritz et al. 2020). Hence, there is a growing evidence-based debate on trade-offs, deleterious effects and related ethical issues regarding biologging of wild-living animals (Vandenabeele et al. 2012; Thaxter et al. 2016; Bodey et al. 2018; Portugal and White 2018).

An alternative approach is to change the frame of reference; not the bird and its equipment is moving in still air, but the air moves towards the bird with flight velocity—hence, the bird appears stationary. This situation can be established using a wind tunnel. Experimental equipment can be placed conveniently aside, and the bird may fly unhindered. As a further advantage, the flight conditions are well reproducible. For that reason, several wind tunnels have been established since the late 1960’s to conduct research with flying birds, see Hedenström and Lindström (2017) for a list of wind tunnels for bird flight research.

Different emphases have been given to wind tunnel research: physiological investigations (metabolic rates, heart rates, oxygen consumption, energy conversion efficiency; see for example Ward et al. (2001, 2004), Hedh et al. (2020), kinematics of bird flight (e.g. Pennycuick et al. 2003) or aerodynamic investigations (flight power, flight range, lift and drag coefficients, detailed velocity distribution via particle image velocimetry (PIV), e.g. Spedding and Hedenström, 2009; Muijres et al. 2012; Johansson et al. 2018). Especially for migration research a wind tunnel is meanwhile indispensable (Hedenström and Lindström, 2017).

Though a wind tunnel represents a very valuable research tool, other issues emerge; birds have to be trained to fly in the wind tunnel, which can be a challenging and elaborate task (Rothe and Nachtigall 1987); and, of course, a wind tunnel with appropriate size and velocity range has to be available. This paper deals with the latter, namely the development and construction of a wind tunnel for bird flight experiments. It covers all aspects of creating such a wind tunnel: layout, design, construction, control, evaluation, up to operation experience. The paper is intended to encourage interested biologists and researchers to create their own wind tunnel. Since only a limited budget was available, many expensive components have been substituted by affordable alternatives, though without affecting the functionality of the final device.

## Motivation

In a European LIFE project (LIFE + 12-BIO AT 000143; Fritz et al. 2017, 2019), a migratory population of the endangered Northern Bald Ibis (*Geronticus eremita*) is reintroduced in Europe. Most research is conducted during the human-led migration flight, where human-raised juvenile birds follow microlight planes (Fritz et al. 2017). Though, in the frame of a research project on the benefits of formation flight in birds, complementary physiological investigations needed to be performed under well reproducible conditions. For that need, a wind tunnel appeared to be the favourable methodological approach. The requirements for the wind tunnel were as follows: the test section should be sufficiently large for that species with a wingspan of approximately 125 cm; it should be permanently available for the time-consuming training and the extensive data collections; it should be transportable to the site of operation, and, as a further constraint, a very limited budget was available. Consequently, we decided to build our own wind tunnel of adequate size which meets all those requirements. In July 2019, the wind tunnel was ready to use and flight training started with a group of four juvenile Northern Bald Ibises.

This paper is about the technical description of the wind tunnel. The data collected with these four birds will be published separately.

## General layout of the wind tunnel

For the layout of the wind tunnel, the size of the birds and their greatest flight speed are fundamental parameters. From human-led migration flights the maximum flight velocity of the species is known to be approximately 50 kmh^−1^ ≈ 14 ms^−1^. To provide enough lateral and vertical space for the birds during their flights in the wind tunnel, an outlet cross section of 2.50 m in width and 1.50 m in height was the design specification.

A closed-circuit wind tunnel (“Göttingen”-type, see Barlow et al. 1999) which exhibits such a big test section would be of enormous length. Extrapolating the data of the Lund wind tunnel (Rosén and Nyström, 1996; Pennycuick et al. 1997) yields a length of 25–30 m. Even if one could cope with such dimensions a building which could house the wind tunnel would have to be available. As a result, experiments would be very expensive. In this particular case neither the budget for such a big wind tunnel nor the housing was available. Consequently, a closed-circuit wind tunnel was out of the question for our requirements.

Recirculating wind tunnels for bird flight research exist up to a cross-sectional area of approximately 1.5 m^2^. For example, Lund wind tunnel (Sweden) and Seekirchen wind tunnel (Germany) have an area of 1.08 m × 1.20 m (Rosén and Nyström, 1996; Engel et al. 2006), and the test section of University Western Ontario (Canada) wind tunnel exhibits 1.00 m × 1.50 m (Gerson and Guglielmo 2011), to mention the greatest published.

Though an open circuit suction wind tunnel (”Eiffel”-type see Barlow et al. 1999) is smaller than a Göttingen-type one, it would be roughly 15 m in length for the required cross-sectional area of the test section. Taking into account the available building to house the wind tunnel, the construction costs and the grade of complexity, the decision was made to create an open circuit blower-type wind tunnel.

A further advantage of a blower wind tunnel is the easy accessibility to the birds. In its open test section, foster parents can be present to efficiently train the birds to fly in the stream. From technical and practical point of view, the accessibility to the motors is also an advantage.

Of course, there are disadvantages of a blower type. The most severe is the inherent higher turbulence level compared to a closed-circuit device, which originates from the upstream fan. We are aware of this fact and tried to laminarize the flow as well as possible.

## Layout and design

As mentioned above, the dimensions of the exit cross section of the outlet are specified to be 2.50 m × 1.50 m. It must be taken into account that the shear layer—the mixing region between the moving air of the jet and the still ambient air surrounding the wind tunnel—increases downstream and reduces the area of laminar flow of the core region, both in horizontal and vertical direction. The maximum estimated flying speed of the birds is 14 ms^−1^; therefore, the design speed of the wind tunnel should be approximately 16 ms^−1^ to have a safety margin.

**Fig. 1:**
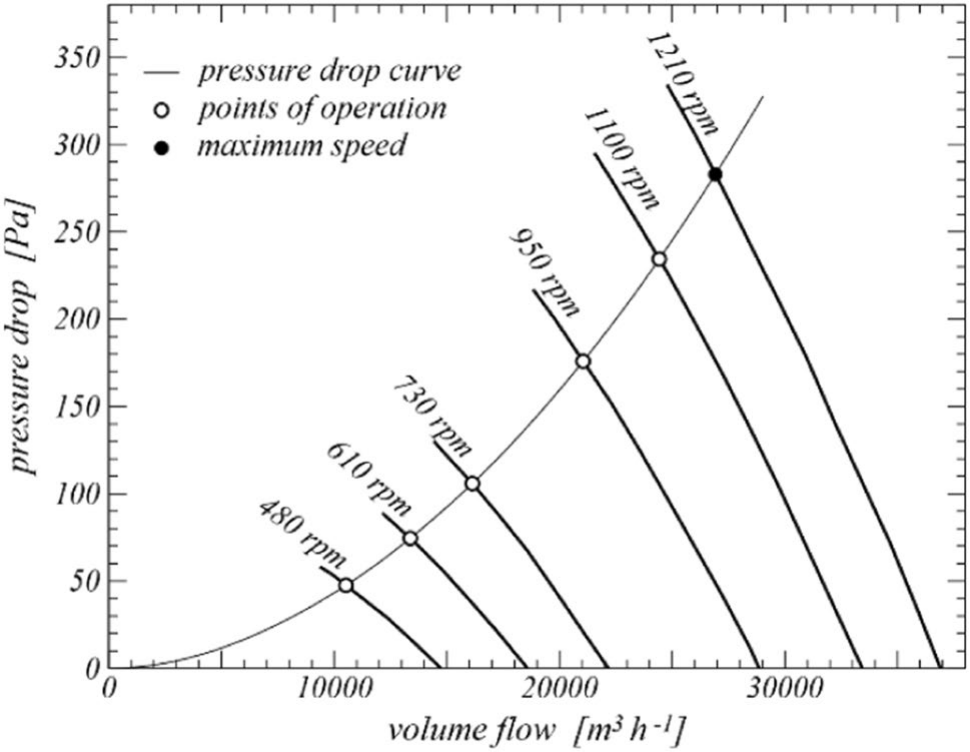
Performance map of one axial fan. Bold solid lines: performance curves for different ventilator speeds; thin curve: pressure loss curve of the wind tunnel. (Performance curves adapted from Ziehl-Abegg 2016)

For the remaining geometrical dimensions of the wind tunnel, the performance of the fan has to be taken into account. Due to dimensional restrictions of the building, a rectangular inlet cross section of 4.30 m in width and 2.15 m in height is designed, yielding an inlet area of

1 $${A}_{1} = 4.30\cdot 2.15 = 9.245\, {m}^{2}$$

with those dimensions, a single fan cannot be implemented. Instead, eight ventilators are installed, which are arranged in two rows of four fans. Each of the ventilators has to provide a volume flow of approximately 27,000 m^3^h^−1^. The fans are chosen according to their pressure-to-volume-flow performance (Fig. 1); in our case, ventilators from a low-noise series by Ziehl-Abegg (2016) fulfil our requirements. The ventilators have a nominal diameter of 91 cm and can be fitted neatly at the inlet side.

**Fig. 2:**
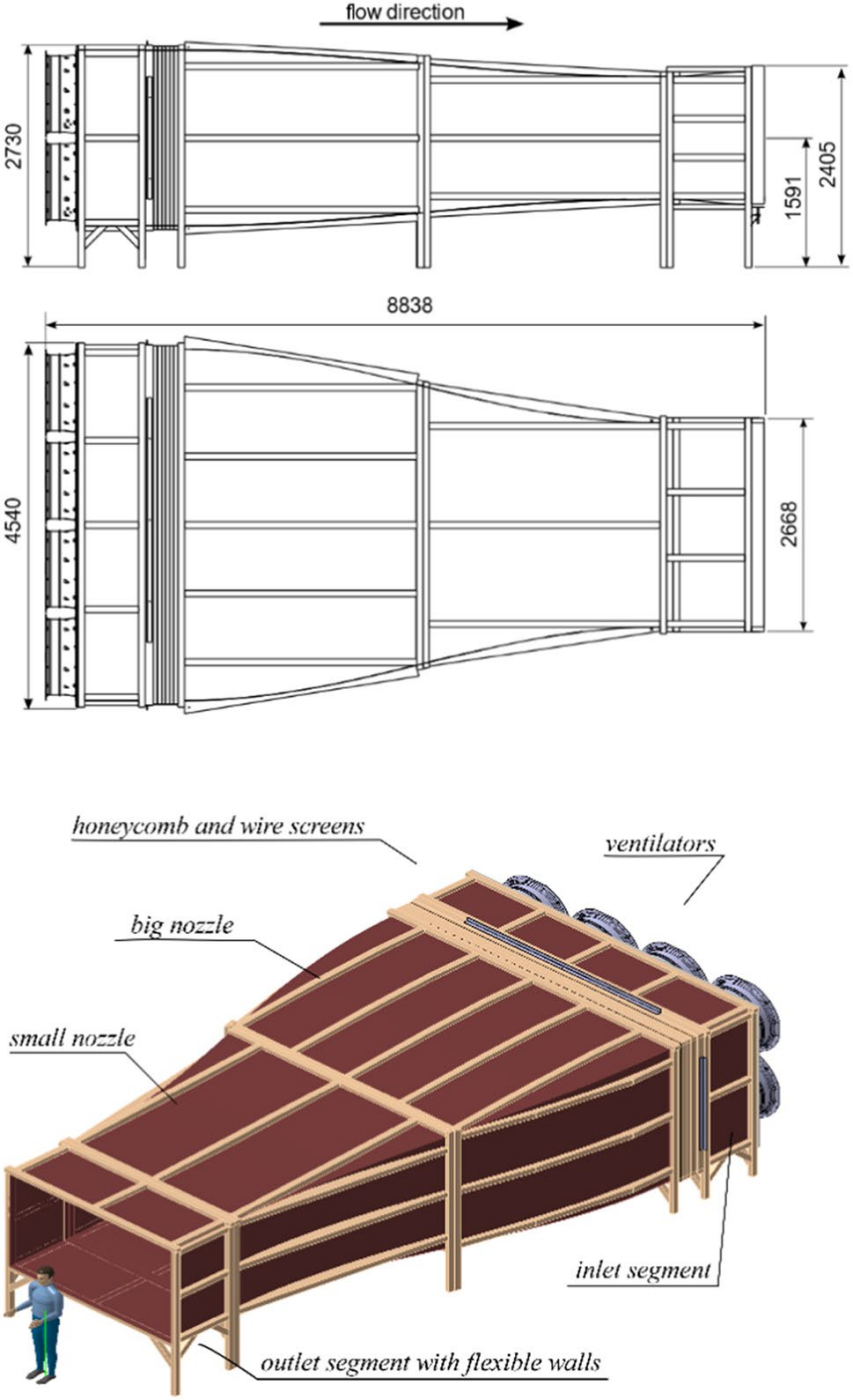
Side view (top), ground view (middle) and axono-metric projection (bottom) of the full wind tunnel. (Dimensions in mm; dummy person in scale.)

The length of the wind tunnel emerges by requiring a smooth curvature of the sidewalls between the inlet and the outlet section to strictly avoid any flow separation, which would induce turbulence and destroy the laminar flow. Generally, a converging nozzle is not prone for separation, but a separation bubble could theoretically establish downstream of the greatest convex curvature. Taking the dimensions of the wind tunnel’s building into consideration, a total length of 8.84 m was conceived, still providing enough space for the birds to fly unhindered (Fig. 2).

Downstream of the eight ventilators a honeycomb is installed, which covers the whole cross section. It extinguishes the unwanted swirl originating from the fans, and aids to direct the flow. Three wire screens downstream of the honeycomb laminarize the turbulent air. The number of screens is a compromise between flow quality in terms of uniformity of the mean flow and turbulence level, and the power requirements.

**Fig. 3:**
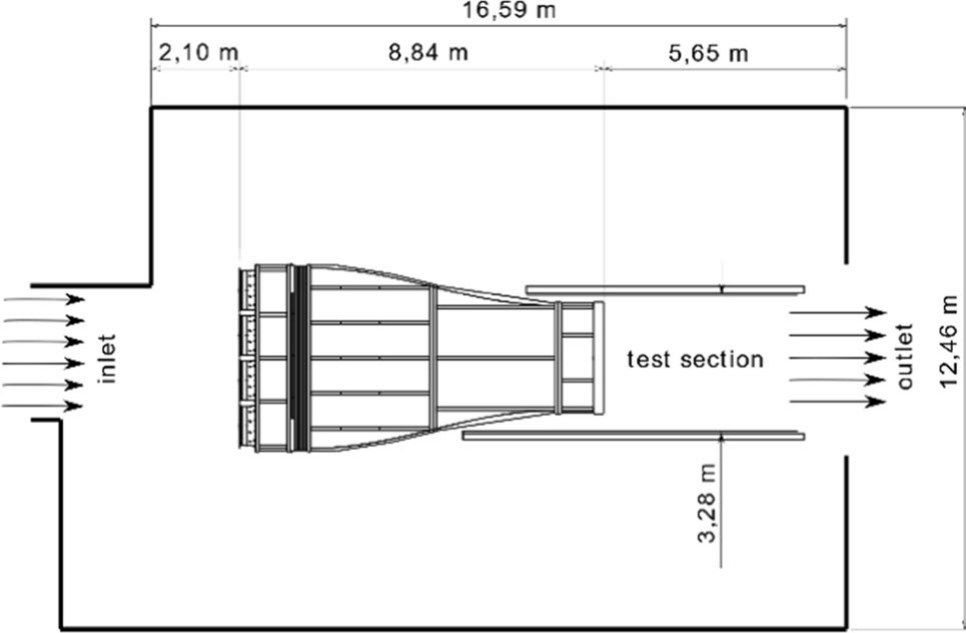
Arrangement of the wind tunnel inside its building

The wind tunnel is positioned in a building which is approximately 16.5 m long, 12.5 m wide and 4 m high (Fig. 3). At the inlet and outlet side of the wind tunnel, the building exhibits openings to the environment. The arrangement is such that air from the environment is sucked in through the inlet and leaves the building downstream of the test section. There is no backflow inside the building.

Though the region downstream of the wind tunnel’s outlet is more than 5 m long, the useful undisturbed airflow extends to approximately 2.5 m. This represents the approximate length of the test section. The test section measures 3.28 m in width, which is wider than the air jet, providing some space for the experimenter and experimental equipment. The outlet of the building is greater than the test section to avoid the air jet impinging onto an edge resulting in recirculations near the test section.

### Pressure losses

To find the operating points of the wind tunnel, the flow resistances of all relevant components have to be known. A flow resistance is formulated in terms of pressure drop *∆p*, which in general is proportional to a characteristic flow speed $v$ squared,

2 $$\Delta p=\zeta \frac{{\rho }_{0}{v}^{2}}{2}$$

with *ρ*_0_ being a reference air density, and ζ representing the resistance coefficient. Each component exhibits a particular resistance coefficient and refers to a characteristic flow speed (Idelchik 1966; VDI-Gesellschaft Verfahrenstechnik und Chemieingenieurwesen 1998). The characteristic flow speed can be expressed in terms of the outlet velocity of the wind tunnel. Adding all pressure losses, a velocity-dependent pressure drop curve is found.

Components relevant for the total pressure resistance are all obstructions along the flow of an air particle. That comprises the entrances of the ventilators, the dust screens and safety grids of the fans, the sudden expansion immediately downstream of the fans, the honeycomb, the laminarization wire screens and finally wall resistance. The resistance coefficients should be regarded as approximations, since the detailed flow situation at the respective component is not known. The contribution to the total pressure loss will be discussed shortly in the following, details are given in the appendix.

### Pressure loss curve

All pressure losses can be evaluated by Eq. (2), with the characteristic velocities being expressed in terms of the outflow velocity, or volume flow, respectively. The pressure drops are summed up to give the total pressure loss for that particular speed. Applying different flow velocities, a velocity-dependent pressure loss curve is obtained (Fig. 1). Several resistance coefficients depend on the Reynolds number. As a consequence, the pressure loss curve is not a polynomial of second order but differs slightly from it. The intersection points of the pressure loss curve with the performance lines of the ventilator represent the operating points for the particular ventilator speeds. The maximum volume flow; thus, maximum velocity is found by the intersection of the pressure loss curve with the performance curve for greatest rotational speed of the fan. A volume flow of $\dot{V}_{s}=26920\hspace{0.17em}{m}^{3}{\mathrm{h}}^{-1}$ for a single ventilator is found, corresponding to a maximum outlet flow speed of

3 $${v}_{2}=\frac{8\hspace{0.17em}\dot{{V}_{s}}}{3600\cdot {A}_{2}}=15.95 \hspace{0.25em} m{s}^{-1}.$$

in terms of equivalent speed. Exemplarily, for that maximum speed, the resistance coefficients, characteristic speeds and pressure losses are listed in Table 1.

**Table 1:** Resistance coefficients, characteristic velocities, and pressure losses for maximum flow speed (15.95 ms^−1^; v in ms^−1^, Δ*p* in Pa)

	*ζ*	*v*	Δ *p*
Dust collector	0.689	11.50	58.87
Safety guard	0.751	11.50	64.18
Entrance	0.540	11.50	46.12
Expansion	0.254	11.50	21.66
Honeycomb	0.211	6.47	5.71
Screen 1	0.898	6.47	24.29
Screen 2	0.898	6.47	24.29
Screen 3	1.025	6.47	27.72
Wall friction	0.012	11.21	10.12
Total pressure losses			282.94

The theoretically achievable maximum speed is slightly below the design speed of 16 ms^−1^, indicating that either the pressure losses are too high, or the volume flow of the ventilators is too small. Since pressure losses can hardly be changed, more powerful fans would have to be used to realize design speed. Nevertheless, as the wind velocity is still above the greatest flying speed of the birds, it was decided to stay with the fans and accept a slightly lower maximum speed. With a further—fourth—screen the pressure loss would increase by 9%, and the design speed could probably not have been accomplished.

It should be noted that the predicted velocity corresponds to standard environmental conditions in terms of pressure (= 101,325 Pa) and temperature (= 288 K) and is therefore referred to equivalent flow speed. The maximum speed of the manufactured wind tunnel was measured to be 15.7 ms^−1^ in terms of true air speed. To compare it with the design speed, it has to be reformulated as equivalent air speed, resulting in

4 $${v}_{2}^{*}\hspace{0.17em}=\hspace{0.17em}{\left(\frac{{\rho }_{2}}{{\rho }_{0}}\right)}^{1/2}\cdot {v}_{2}\hspace{0.25em}=\hspace{0.25em}15.4\hspace{0.25em}m{s}^{-1}$$

with *ρ*_*2*_ = 1.17 kgm^−3^ being the ambient density during the measurement, and *ρ*_*0*_ is the reference density corresponding to the mentioned reference state. Considering that several assumptions and approximations have been used for the calculation of the pressure losses, the evaluation of the maximum flow speed is quite good, the error is less than 3.5%.

## Numerical simulation of the flow

**Fig. 4:**
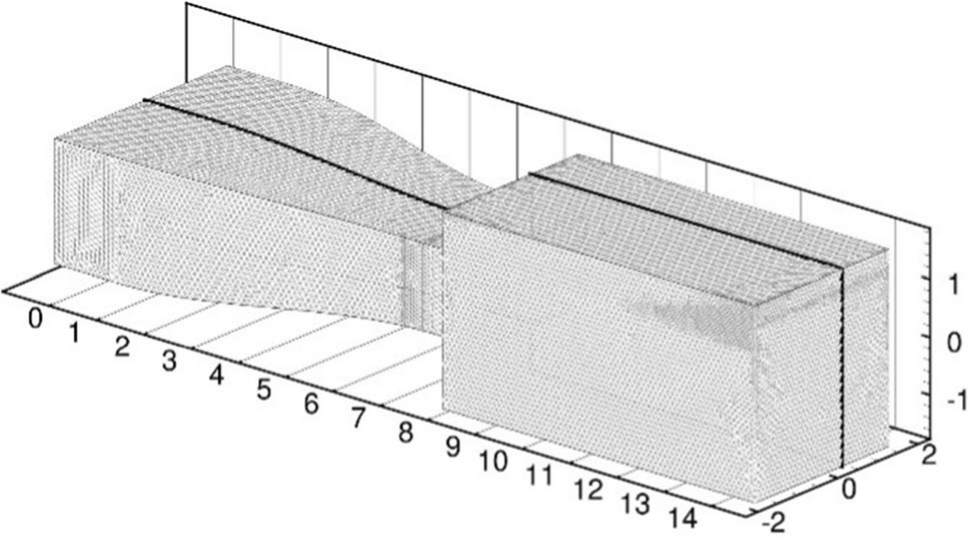
Computational mesh for the flow simulation. Dark line along the domain denotes the plane for streamline visualization of Fig. 5

During the design process, several numerical calculations have been performed to evaluate the flow situation downstream of the outlet cross section using the computational fluid dynamics module of Siemens NX (Siemens 2016). To prevent the birds to fly out of the testing area, side walls have been installed 0.5 m and 0.2 m apart from the free stream. Numerical simulations showed that those walls can influence the direction of the free stream exiting the wind tunnel. The calculation grid is shown in Fig. 4. It consists of the wind tunnel and the complete flow domain downstream of the outlet cross section. Different arrangements of closed walls near the outlet have been assessed. It turned out that the sidewalls can change the flow by sucking air from the top and redirect the free-stream flow downward. By means of several numerical simulations it was found that closing the sidewalls and the top gives an undisturbed free stream.

**Fig. 5:**
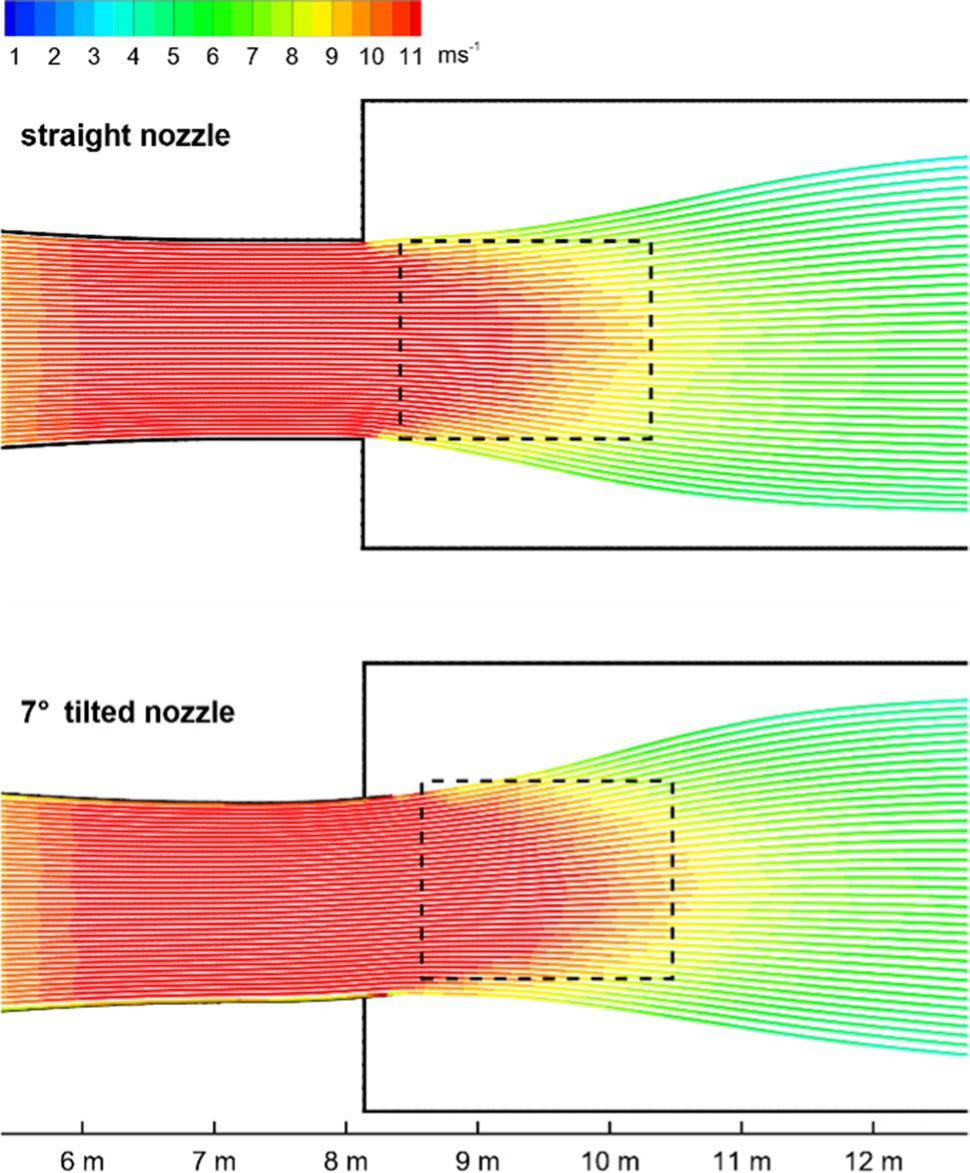
Streamlines in a vertical cut through the center of the wind tunnel. Colour represents absolute velocity. Top: straight nozzle, bottom: tilted nozzle in extreme upward position. (Dashed rectangle denotes approximate flying region of the bird.)

Several horizontal slopes of the outlet nozzle have been designed in the numerical model to direct the flow. The streamlines of the nozzle in 0° position and with maximum inclination of 7° are presented in Fig. 5. For the approximate flight region, marked by a dashed rectangle, the velocity distribution is very even and directed horizontally for the straight nozzle. A close inspection of the streamlines of the tilted nozzle shows the slight upward bend of the nozzle’s exit, which generates an updraft. For the shown 7° adjustment a horizontal and vertical speed of approximately 11 ms^−1^ and 1.2 ms^−1^ is achieved, respectively. As an estimate, a glide ratio (L/D ratio) of 9:1–10:1 emerges for this flow conditions, which appears reasonable for that species. Training flights showed that the birds could glide in this kind of flow. The observed flying position of the bird is in the center of the free stream or higher, which is roughly 150–180 cm above ground, hence more than the span of the species. For that, any ground effect—known from landing aircraft—will not influence the bird’s flight performance and therefore need not to be considered.

## Construction

The wind tunnel is made up by four segments to facilitate manufacturing and transportation to the site of operation (Fig. 2):

1. rectangular inlet segment taking up the fans (length = 84 cm);
2. big concave converging nozzle (300 cm);
3. small convex converging nozzle (300 cm);
4. rectangular outlet section with flexible horizontal walls (120 cm).

The honeycomb and the three wire screens are positioned downstream of the ventilators, between the first and the second segment. They are designed as frames carrying the particular component and can be clamped together to make up an additional package.

The outlet segment exhibits flexible walls at the bottom and on the top. They can be adjusted in parallel to direct the stream upward (maximum + 7°) or downward (− 5°) to provide wind for gliding and climbing flights. Each segment exhibits the same construction technique; a strong wooden frame is covered with smooth panels which are glued onto the frame, making up the inner walls of the wind tunnel. Each part of a segment—sidewalls, top and bottom element—is detachable, so that only flat elements constitute the wind tunnel, except the walls of the nozzles, which are slightly curved.

The wooden construction has many advantages. It is easy to tool with no expensive machines being necessary. Due to its rather low weight, it can easily be handled in the workshop and for transportation. Despite its great dimensions, the individual elements of each segment can be lifted and carried by two persons. Further, the costs of a wooden construction are much lower than those of a metal one, for example.

As a drawback, the sensitivity of a wooden structure to humidity has to be mentioned. Since wood has the tendency to deform in humid conditions, water-resistant panels and bonded wooden rods are used. During one and a half years of operation under different ambient conditions, only marginal deformations can be observed, which do not affect the performances of the wind tunnel.

All parts have been constructed at University of Applied Sciences Joanneum, and subsequently dissembled and transported for about 300 km to the site of operation, where the bird’s camp is located. There, the wind tunnel was reassembled.

## Electrical equipment and controls

Due to the cost aspect, a cheap and reliable solution for the control was investigated as well. Each ventilator is equipped with a factory mounted frequency converter and control unit, and has a power supply of 400 V and a maximum current of 6 A. To start the ventilator a 24 V pilot contact has to be closed. Once the contact is enabled the speed of the ventilators can be controlled by an analog signal between 0 and 10 V, representing the rotational speed of the ventilators. This analog signal is adjusted using a potentiometer.

The flow velocity is measured by a small, calibrated vane anemometer (Schiltknecht 2016a) of 22 mm in diameter, which is located at the top of the outlet segment, reaching approximately 20 cm into the stream. It converts the actual rotational speed of its vane into a linear voltage signal, which can be processed and displayed as flow velocity. The measuring range is from 0.4 to 20 ms^−1^ with an accuracy of 1% error of the full range and 1.5% error of the reading, respectively (Schiltknecht 2016b). The response time is 1.0 s for increasing and 8.0 s for decreasing velocity. Since the wind tunnel is run in steady mode, the response time of the anemometer does not affect the operation of the wind tunnel.

Several sensors measure the ambient absolute pressure, air temperature and humidity to evaluate the actual density of the air, which is necessary to transform the measured true air speed to equivalent air speed. The density is calculated via the ideal gas law, see appendix.

All sensor signals and the motor signals are connected to a microcontroller. This central control unit is realized using an Arduino Mega, which is very reliable, easy to program and inexpensive (Schmidt 2015). The microcontroller offers an I^2^C interface for digital input (pressure, temperature, and humidity sensors), and several channels for analog signals (anemometer signal). Incoming signals are low pass filtered. Further, digital moving average filter is applied to smoothen the signals and get rid of any superimposed disturbances.

The microcontroller is programmed to generate eight pulse width modulated outputs to control each single ventilator. Those outputs are subsequently converted into voltage with four dual full bridge drivers. The speed is adjusted for all ventilators together, but the arrangement is such that the program allows to trim and offset the speed of each ventilator separately. Also, the pilot contacts of each ventilator are controlled individually to get information, if there is any malfunction. Since the reaction time of the fans is quite high, the filtering of the signals does not represent any problem for the control algorithm.

With this setup it is possible to control the powerful ventilators with very cheap, but accurate devices. The 5 V based Arduino Mega is able to control the whole setup with a voltage of 400 V and 48 A current, resulting in a total power of 32 kW.

A control box containing the microcontroller, the wiring and the sensor cables is located left of the outlet of the wind tunnel, so that it can be operated in the vicinity of the flying birds. It comprises an LCD display to indicate relevant data like the current equivalent air speed, temperature, and density.

Furthermore, a large seven-segment display is installed on top of the wind tunnel’s outlet to inform the foster parent about the actual speed during the flight.

All relevant data are stored on a micro-SD card with 1 Hz as ancillary data for the physiological experiments.

## Measurement of the flow quality

**Fig. 6:**
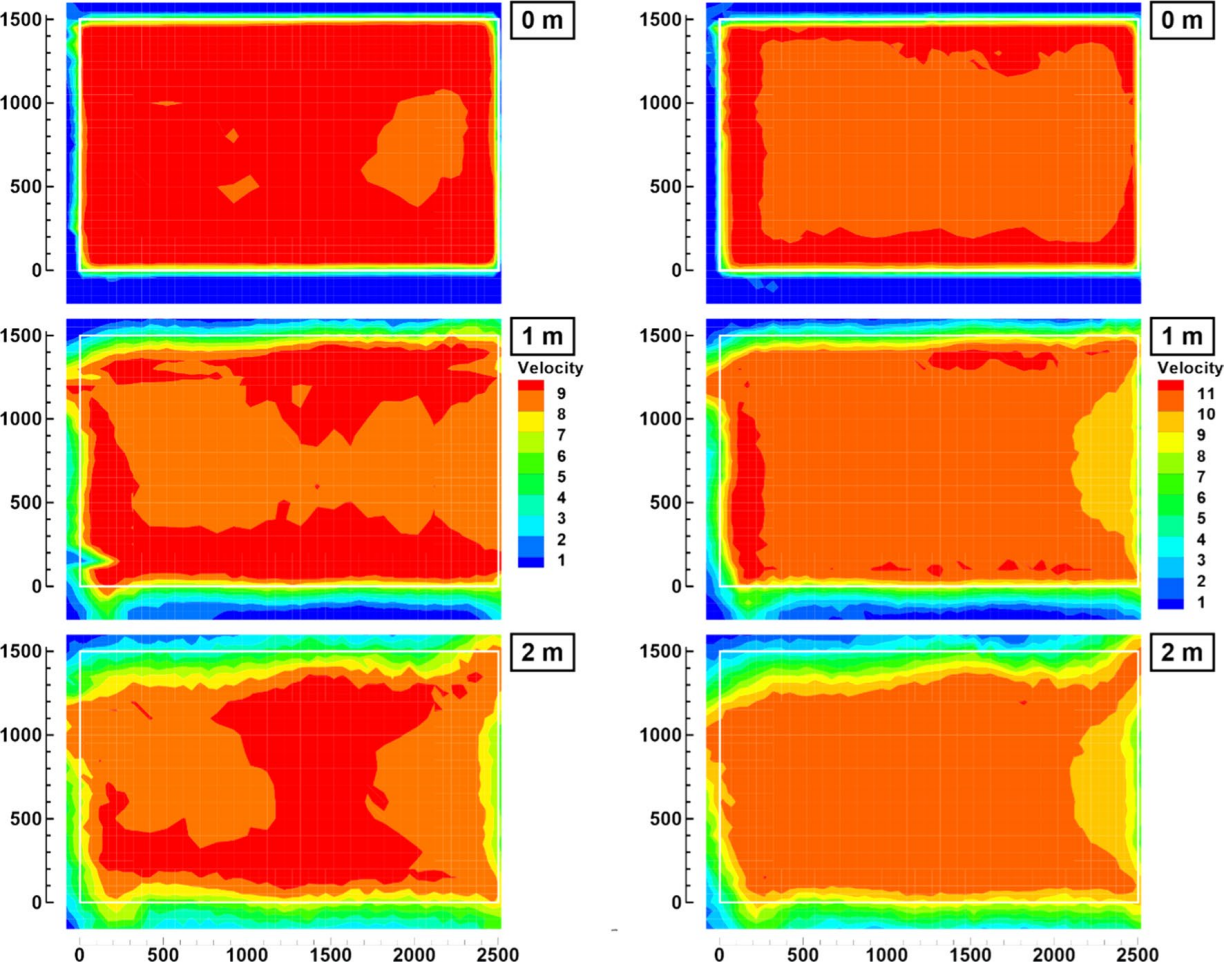
Measured mean velocity distribution for a nominal speed of 9.15 ms^−1^ (left) and 10.70 ms^−1^ (right) at different planes perpendicular to the flow. Streamwise positions: direct at outlet (top), 1 m (middle), 2 m (bottom); (Velocity in ms^−1^, white rectangle denotes the exit cross section. View is upstream, as seen by the flying bird.)

The velocity distribution was evaluated using the same vane anemometer which is implemented in the wind tunnel during regular operation. A specially designed and constructed device automatically measured the velocity distribution in prescribed planes perpendicular to the flow direction and stored the data. For two representative flight velocities of the birds, namely 9.15 ms^−1^ and 10.7 ms^−1^, the velocity distribution in three different planes was measured. The first plane is located directly at the outlet, whereas the second and third planes are located one and two meter downstream, respectively. This corresponds roughly to the flight position of the birds. Each plane exhibits 1450 measurement points, giving a good overview of the mean velocity distribution (Fig. 6). Due to its response time the vane anemometer was held in the stream for a few seconds to allow the vane to adjust. Subsequently, the velocity was measured for 10 s and timely averaged.

Apparently, the flow is very even. The wake of the electric motors, which are mounted in the center of the ventilators, do not appear in the velocity distribution. At a downstream position of 2 m, the mixing layer of the free stream with ambient air exhibits a thickness of approximately 30 cm. This position represents the end of the useful free jet of the open test section. Further downstream the healthy core flow becomes too small for the birds to fly in the laminar stream; turbulence due to mixing are too strong to make reproducible results possible, though it was found that the birds are able to fly in that region as well.

**Fig. 7:**
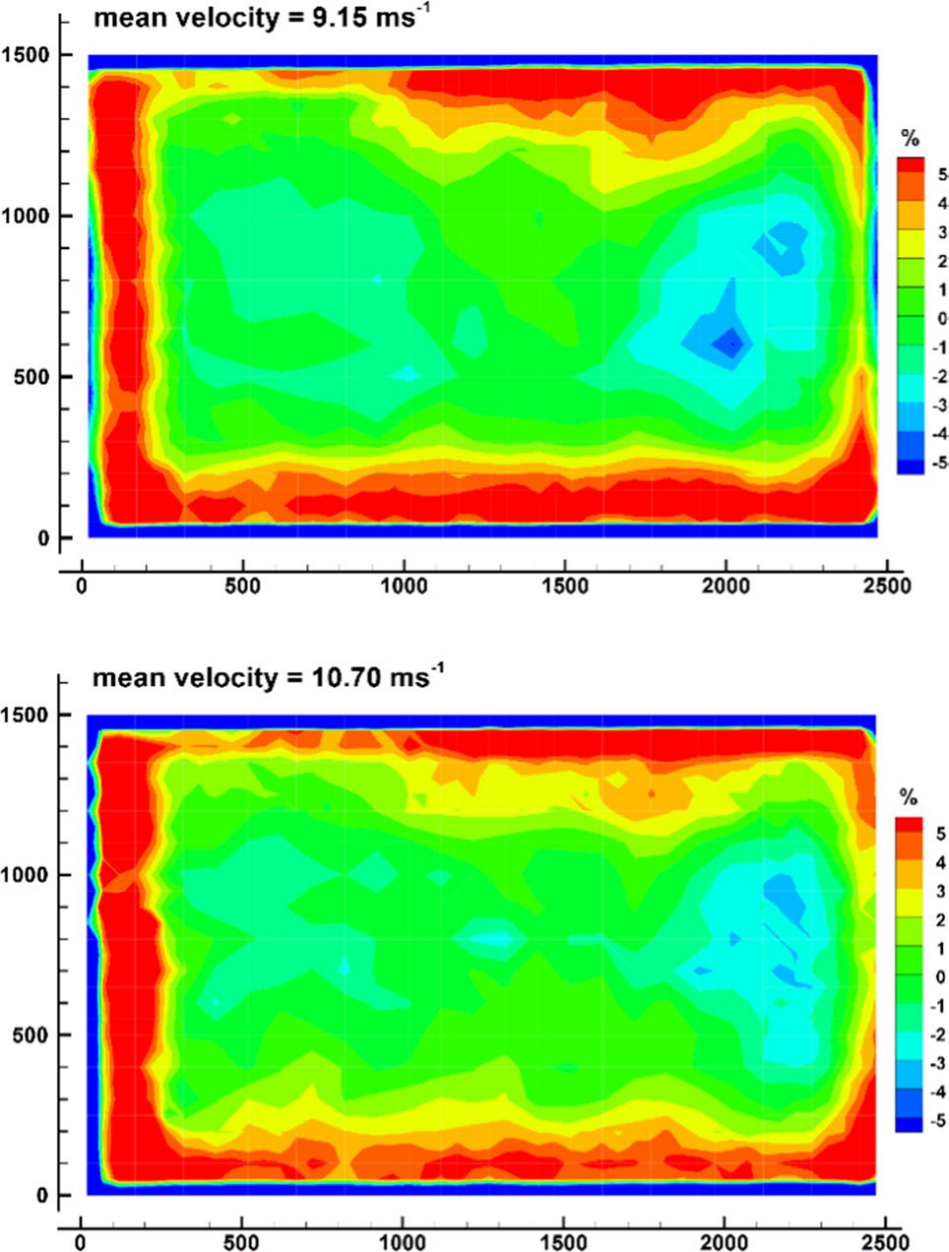
Percental deviation of local mean wind speed from overall mean velocity 9.15 ms^−1^ (top) and 10.70 ms^−1^ (bottom) at the exit cross section. (The area shown corresponds to the white rectangle of Fig. 6)

The deviation of the local flow speed from the mean velocity averaged over the whole outlet cross section is presented in Fig. 7 for both speeds. Apparently, the velocity is about 5% higher in the wall region, up to 20 cm off the boundaries. Due to the curvature of the geometry the near-wall layers encounter a stronger pressure gradient than in the core region. Consequently, they undergo a greater acceleration than in the center of the wind tunnel. Inside the core region, the deviation is remarkably small, mostly less than 2% of the mean flow speed. A close inspection shows a region of slightly smaller velocity at the right boundary. It is suspected that it originates from unsymmetric inflow conditions, since two ventilators at that particular side are located behind a corner of the building. The speed of the two particular ventilators has been increased, but apparently did not yet fully eliminate the deficiency.

### Turbulence intensity

The turbulence intensity of the flow was assessed using hot-wire anemometry. The measuring principle is based on the changing electric resistance of a metal wire when exposed to different flow velocities. The wire of the sensor is extremely small to exhibit minimal thermal inertia and to establish potential flow conditions around the wire. A special electronic circuit regulates the voltage to keep the temperature of the wire constant. Those changes in voltage are a very accurate and dynamic measure for the actual flow velocity, once the hot-wire sensor has been calibrated. For the current application, a constant temperature anemometry (= CTA) equipment of Dantec Dynamics A/S was used (Jørgensen 2002). The one-dimensional sensor of type 55P11 comprises a platinum plated tungsten wire with a length of 1.25 mm and a diameter of 5 μm. Placed normally to the flow it is able to scan the actual velocity with several kHz. At each particular measurement position, *n* = 1024 samples were taken with a sampling frequency of 1 kHz. The analogue data is digitized and processed using a software (Mini-CTA) of the aforementioned vendor. The velocity fluctuations $u^{\prime}$ with respect to the timely averaged mean velocity *U* represent the turbulent fluctuations at the current position. CTA measurements of the flow velocity—hence turbulent fluctuations—have been performed the same planes as the mean-flow measurement given above. Measurements were taken at equidistant positions, with a spacing of 20 cm both horizontally and vertically, respectively, resulting in 13 times eight points for each plane. For the measurements the support of the sensor has been mounted on a stable tripod to avoid any movement or oscillations of the sensor, which would introduce erroneous velocity fluctuations. For the mean velocity 9.15 ms^−1^ was chosen.

**Fig. 8:**
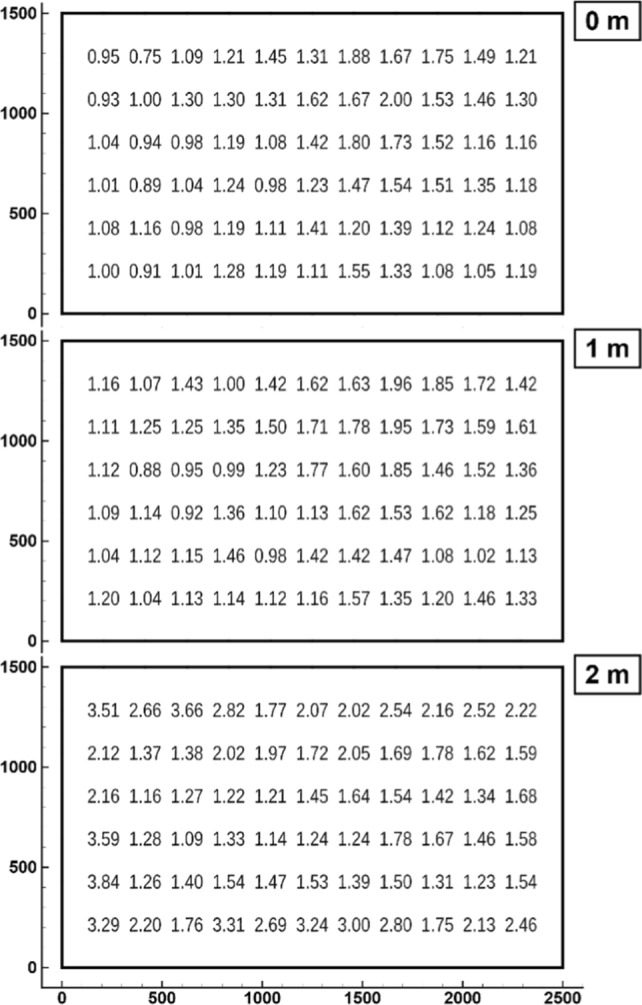
Turbulence intensity at the same planes as Fig. 6 for mean velocity 9.15 ms^−1^ and 0° inclination. (Values are in percent; the area shown corresponds to the white rectangle of Fig. 6; view is upstream)

The turbulence intensity Tu is an adequate quantity to describe turbulence in a flow. It is defined as the root-mean-squared value ${u}_{rms}$ of the turbulent fluctuations ${u}^{\prime}={u}_{i}-U$ related to the local mean velocity $U$, in

5 $$Tu=\frac{{u}_{rms}}{U},\hspace{1em} with\hspace{1em} {u}_{rms}=\frac{\sqrt{\sum {\left({u}_{i}-U\right)}^{2}}}{n-1}$$

where ${u}_{i}$ represents one of the *n* actually measured velocity values at the current position. The turbulence intensity is presented in Fig. 8 for all three planes and for 0° inclination. The readings of the mixing layer at the edge of the free jet have been omitted since they do not represent the flow in the test section. Inside the flow region, the turbulence intensity is approximately 1% at the exit cross section and up to 2% at the end of the test section, which appears to be acceptable, though these are rather high values compared to wind tunnels specially designated for aerodynamic research. To reduce the turbulence level additional wire screens with finer meshes would have to be implemented, though with these additional flow resistances the required flow velocity could presumably not be achieved with the installed fans. For that reason, the turbulence level has been accepted as it is.

## Manufacturing costs of the wind tunnel

Though costs are not the primary scientific interest of a wind tunnel, they are listed in the following to give the interested reader an estimate of the necessary budget, see Table 2. All in all the material sums up to approximately € 25.000. Work time has not been taken into account since it depends on the local availability of craftsmen or skilled volunteers (students). Totally, about 800 h of working time were necessary to design and manufacture the wind tunnel, which took place within five months. The costs of the tools are not taken into account, since they depend on the workshop’s equipment. It has to be added that no special machine tools are necessary; all the used tools can be acquired for less than € 2.000, if the project is started from scratch.

**Table 2:** Material costs of the wind tunnel (in €)

Eight fans	12.000,-
Wood for frames	2.000,-
Wood for panels	1.000,-
Three wire screens	2.500,-
Honeycomb	1.800,-
Electronic components	400.-
Anemometer	800.-
Electric components (fuses, switches)	1.000.-
Wires and cables	500.-
Housing for electric components	1000.-
Cutting curved wooden rods	1.200.-
Small parts	500.-

Depending on how manpower is rated, the total costs are five to ten percent of a commercially built closed-circuit wind tunnel.

Operating costs comprise the rent for the building and electrical power. The demand on electric energy at full speed is 32 kWh per hour. Costs for maintenance can be neglected, since repairs or changes can be made by the operator.

## Discussion

Despite the many advantages an open wind tunnel has, there are also drawbacks. As already mentioned, the turbulence level of a blower type wind tunnel is higher than for an Eiffel-type (suction), or a Göttingen-type (closed circuit) wind tunnel. However, the Northern Bald Ibises coped well with the turbulence level and exhibit a high willingness to fly. We assume that the aerodynamic performance of this large size birds is hardly affected by small scale turbulence, the inherent higher Reynolds number due to the bigger size and greater flight speed makes the large bird more resilient for separation, hence more resistant to sudden changes in drag. Further, it is assumed that a flying bird can be regarded a slender body, which is not prone for flow separation. Small birds are more sensitive in that respect (Ortega-Jimenez et al. 2016). Of course, the turbulence level in a wind tunnel should be as small as possible, but the absolute level appears to be of secondary importance, since trends can be evaluated anyhow. It is primarily important that the flight conditions in the wind tunnel are constant and reproducible, and this is the case. This is in contrast to natural flight conditions where always a certain amount of turbulence is present, whose intensity is generally unknown and cannot be controlled.

An open-circuit wind tunnel is prone to collect dust on the wire screens, since it sucks the air directly from the environment. To prevent contamination dust screens have been installed to protect the built-in components, which proved very efficient.

During operation, the building’s gates are open to provide the high volume flow, which is sucked in by the eight fans, and at the same time is blown out at the exit cross section. This implies that meteorological conditions have to be taken into account, i.e., changing temperature, ambient pressure and humidity. Since experiments are based on equivalent air speed, those parameters are factored in and do not influence the collected data. Nevertheless, the bird’s reaction on a changing atmosphere is difficult to foresee and no training or data collection was done in high humidity condition or at temperatures below 10 °C.

As a consequence of the wind tunnel sucking ambient air, the local wind conditions have to be taken into account. If the wind is strong or gusty, measurements are not reliable since the air speed may change faster than the volume flow of the fans can be adjusted. The impact of the atmospheric conditions on the wind tunnel is due to the restricted dimension of the building which houses the wind tunnel. In a sufficiently large housing, the wind tunnel could be operated as a closed system, independent of atmospheric conditions.

A movable perch has been installed to indicate the end of a flight. The perch can be erected remotely when the training flight or the scientific experiment is finished. During the flight, it is retracted flat on the floor.

The noise of the wind tunnel during operation is rather low, not least due to the ventilators, which are designated low noise. Nevertheless, the birds have to get accustomed to it.

Practical experience could be gained in 2019 and 2020 during flight training for scientific experiments. Four Northern Bald Ibis chicks were human-raised particularly for that purpose. Training methods comprised socio-positive interactions with the foster parents using gradual habituation and operant conditioning (Zeligs 2014). Already during hand-raising, the chicks were regularly exposed to playback of the wind tunnel sound for habituation. The gradual habituation of Northern Bald Ibis to a certain sound—a microlight aircraft—has already been successfully practised during training for the human-led migration (Fritz et al. 2019).

**Fig. 9:**
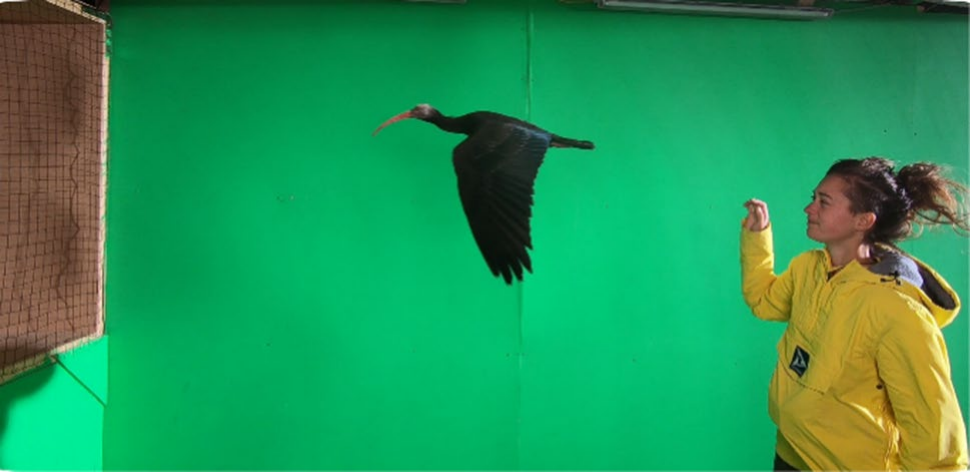
One of the four hand reared Northern Bald Ibis flying in the free stream of the wind tunnel, foster parent downstream

Wind tunnel flight training started about one month after fledging and lasted for the rest of the season 2019. Since the test section is designed wider and higher than the exit cross section of the wind tunnel, the foster parent can stay with the bird during operation of the wind tunnel but remains outside of the flow region (Fig. 9). The presence of the foster parent facilitates flight training.

Care must be taken for the flying bird not to move out of the flow region: as soon as it moves out of the wind, the relative velocity to the ambient air is zero. As a consequence, there is no lift to support the bird, which poses a risk of crash landing and injury. For this reason, side walls have been installed right (0.5 m) and left (0.2 m) of the free-stream to prevent the bird to incidentally fly out of the wind. We noticed that the birds sense the mixing region and quickly learned to avoid flying into the turbulent shear layer.

The wind tunnel was constructed with the aim to conduct physiological and behavioral measurements. For a first experiment birds were fitted with respirometry masks with an air tube running from the mask to an oxygen analyser to measure oxygen consumption at varying flight speed. For this setup an experimenter was standing in the wind tunnel alongside the flying bird, holding and manually adjusting the tube.

## Conclusion

A blower type open circuit wind tunnel for bird flight experiments has been designed, built and evaluated. In the authors’ opinion, an open circuit wind tunnel is the most reasonable option for flight experiments with birds having a wingspan of more than a meter, both for technical and financial reasons. It turned out that rather simple methods were sufficient to design the wind tunnel based on its desired speed and outlet cross section. Evaluation showed that the desired maximum speed has been achieved, and that the velocity distribution at the outlet cross section deviates less than 2% of the averaged outflow velocity. The turbulence intensity within the test section was measured to be between 1 and 2%, which is a reasonable, but not a very low value.

The used material, wood, is well suited for the construction and ensures reliable functionality, at least over a period of two seasons. Long-term experience is not yet available, but according to the condition of the device after the experiments, the authors assume continued functionality over several further seasons. The wind tunnel is intended to perform physiological and behavioral experiments; therefore, a perfect aerodynamic characteristic was not of primary importance.

It was found that technically complex features, for example an acoustic optimization or visual projection of landscapes, are not necessary for the birds to fly in the artificial wind. It rather is important to accustom the birds as soon as possible to the noise and the flow of the wind tunnel. However, the bird training prior to flight experiments requires a considerable amount of time.

The decision to design and construct a wind tunnel ourselves has proved true. The available budget was extremely small in comparison with the building costs of a closed-circuit wind tunnel, being approximately five to ten percent. The close interdisciplinary cooperation between engineers with expertise in fluid dynamics and biologists with experiences on behaviour and physiology of the species proved very fruitful. From a biological perspective, the wind tunnel fulfils the objective to provide a complementary methodological approach for research on physiology of bird flight under controllable and reproducible conditions.

The authors hope that their experience with the low-budget wind tunnel will also inspire other working groups to create their own wind tunnel; the authors have no commercial interests and are willing to share their technical and biological experiences.

## Supplementary Information

Below is the link to the electronic supplementary material.

Supplementary file1 (DOCX 27 KB)
