# Supplementary material for: A low-cost wind tunnel for bird flight experiments
Source: J Ornithol. 2022 Jan 19;163(2):599–610. doi: 10.1007/s10336-021-01945-2 (PMC8993750; doi:10.1007/s10336-021-01945-2)
Supplement: Supplementary file 1 — Supplementary file1 (DOCX 27 KB) [file 10336_2021_1945_MOESM1_ESM.docx]

**Electronic Supplementary Material**

**A low-cost wind tunnel for bird flight experiments**

**Herwig A. Grogger ^[[1]](#footnote-1)^🖂 ^[[2]](#footnote-2)^ · Martin Gossar 1 · Michael Makovec 1 · Johannes Fritz ^[[3]](#footnote-3)^ ·Katharina Neugebauer 2 · Frederik Amann 2 · Bernhard Voelkl ^[[4]](#footnote-4)^**

University of Applied Sciences Joanneum, Engineering Department, Alte Poststrasse 149, 8020 Graz, Austria

^2^ Waldrappteam, 6162 Mutters, Austria

^3^ Animal Welfare Division VPHI, University of Bern, Switzerland

Corresponding author: HAG: ++43-316-5453-8457, herwig.grogger@fh-joanneum.at

**Pressure losses of components**

Pressure loss of the entrances

Resistance coefficients for entrances with a certain distance from the mounting wall are given by Idelchik (1966, section III, nb.1, page 92). The experimental data suggest a constant resistance coefficient

| $\zeta= 0.540 ,$ | ($SEQ Equation \backslash* ARABIC$ $1$) |
| --- | --- |

based on their geometrical data of the implemented ventilators,

$$\frac{b}{D_{h}}=0.42\quad\text{and}\quad\frac{\delta_{1}}{D_{h}}=0.04 .$$

The corresponding characteristic velocity is intake velocity of the ventilators.

Pressure loss of the safety grid

The safety grid in front of each ventilator protects the moving fans from intrusion of objects. It is a strong metal grid, which exhibits considerable pressure resistance. An expression to estimate the resistance coefficient of a grid is given by Idelchik (1966, section VIII, eq. (8-10), p. 308),

| $\zeta=k_{0}\left( 1-f \right)+\left( \frac{1}{f}-1 \right)^{2} ,$ | ($SEQ Equation \backslash* ARABIC$ $2$) |
| --- | --- |

with *f* denoting the ratio of open to total area of the grid. For Reynolds-numbers *Re* > 400 and smooth wire, $k_{0}=1$. The dimensions of the used safety grid yield *f* = 0.621, which results in a constant resistance coefficient

| $\zeta= 0.751 .$ | ($SEQ Equation \backslash* ARABIC$ $3$) |
| --- | --- |

As characteristic velocity the flow in front of the grid is defined.

Pressure loss of the dust collector and the wire screens

A flyscreen made of thin threads is spanned over the inlet of each ventilator. The resistance coefficients both of the dust screens and the wire screens can be computed using eq. (6), though $k_{0}=1.3$, see Idelchik (1966, section VIII, eq. (1b), p. 327). For Reynolds-numbers *Re* < 400, based on wire diameter and inflow velocity, the ζ-value must be adjusted using a Reynolds-number dependent correction factor $k_{Re}$ ,

| $\zeta_{Re}=k_{Re} \zeta,$ | ($SEQ Equation \backslash* ARABIC$ $4$) |
| --- | --- |

which is determined from experimental data, (ibid., p. 327). Hence, the resistance coefficients of the dust collector and the wire screens depend on Reynolds-number, consequently, on velocity. Dimensions of the dust collector and the three wire meshes are given in Table S1; ζ-values apply for maximum flow velocity. The first and the second wire screen exhibit the same parameters, whereas the third one is finer.

**Table S1** Data of dust collector and wire screens (mesh size and wire diameter in mm).

|  | mesh  size | wire  diam. | *f* | *Re* | *k_Re_* | *ζ_Re_* |
| --- | --- | --- | --- | --- | --- | --- |
| Dust collector | 1.30 | 0.28 | 0.677 | 231 | 1.064 | 0.689 |
| Screen 1 & 2 | 2.10 | 0.56 | 0.623 | 260 | 1.050 | 0.898 |
| Screen 3 | 1.72 | 0.50 | 0.600 | 232 | 1.064 | 1.025 |

The corresponding characteristic velocity for the pressure drop of the dust collector is inlet speed of the ventilators. For the wire screens, the speed upstream of them applies.

Pressure loss of the sudden expansion

When the flow exits the circular housing of the fans and subsequently enters the first segment of the wind tunnel, a pressure loss due to this sudden expansion occurs. For symmetric flow it can be determined analytically, though Idelchik (1966, section IV, eq. (4‑3), p. 114) proposes a formula, which also accounts for unsymmetric flow into the expansion,

| $\zeta=N\left( 1-\frac{2}{3n} \right)+\frac{1}{n^{2}}+\frac{1}{3n} ,$ | ($SEQ Equation \backslash* ARABIC$ $5$) |
| --- | --- |

where *n* denotes the expansion ratio, and *N* is the factor accounting for non-uniformity of the inflow. For $N=1$ a uniform flow into the expansion would be modelled; in the current case the inflow is certainly not uniform, though its grade is unknown. Therefore, $N=1.1$ is chosen, which probably underestimates the skewness of the flow. Hence, the constant resistance coefficient

| $\zeta= 0.254$ | ($SEQ Equation \backslash* ARABIC$ $6$) |
| --- | --- |

emerges, with the characteristic velocity being defined as the speed through the fans.

Pressure loss of the honeycomb

An empirical formula to estimate the pressure loss in a hexagonal honeycomb is given by Barlow et al. (1999, eq. 3.43),

| $\zeta=\lambda\left( 3+\frac{l_{0}}{d_{h}} \right)f^{2}+\left( f-1 \right)^{2} .$ | ($SEQ Equation \backslash* ARABIC$ $7$) |
| --- | --- |

with *λ* denoting a surface friction coefficient, *f* being the ratio of open to total front area, *l_0_* is the dimension of the honeycomb in flow direction, and dh denotes the hydraulic diameter of one cell. With estimated values $\lambda=0.026$ and $f=0.95$, and a length to diameter ratio $l_{0}/d_{h}=6$ of one honeycomb cell, a resistance coefficient

| $\zeta= 0.211$ | ($SEQ Equation \backslash* ARABIC$ $8$) |
| --- | --- |

emerges, which is similar to the empirical value of 0.20 proposed by Barlow et al. (1999) for hexagonal honeycombs. As characteristic velocity the speed upstream of the honeycomb is defined.

Pressure loss of the walls

Since the inner surface of the wind tunnel does not represent a simple geometry for which resistance coefficients are listed, the ζ-value of a flat wall is used to estimate the pressure drop of bottom and top wall and the sidewalls, respectively. Since the curvature of these walls is very small, a flat wall is a very good approximation (VDI-Gesellschaft Verfahrenstechnik und Chemieingenieurwesen (1998), section Lb1, eq. 6). The resistance coefficient again depends on the Reynolds- number,

| $\zeta=0.0054+\frac{0.3964}{Re^{0.3}} ,$ | ($SEQ Equation \backslash* ARABIC$ $9$) |
| --- | --- |

for$2\times{10}^{4}<\mathrm{Re}<2\times{10}^{6}$, with the Reynolds number based on the mean hydraulic diameter. Due to the varying cross-section and the corresponding changing flow velocity in the wind tunnel, the mean velocity in the wind tunnel is used as characteristic velocity. For maximum flow speed

| $\zeta=0.012 .$ | ($SEQ Equation \backslash* ARABIC$ $10$) |
| --- | --- |

Calculation of the density of humid air

Humidity can change the gas constant of air by a few percent. Using measured values for ambient pressure *p*, temperature *T* and relative humidity φ, the density of humid air can be calculated as a mixture of dry air and water vapour,

| $\rho=\frac{p M_{a}}{R_{m} T}\left[ 1-\varphi\frac{p_{s}}{p}\left( 1-\frac{M_{w}}{M_{a}} \right) \right] ,$ | ($SEQ Equation \backslash* ARABIC$ $11$) |
| --- | --- |

where $R_{m}$ is the universal gas constant, $p_{s}$ stands for the saturation pressure and $M_{a}$ and $M_{w}$ are the molar mass of dry air and water, respectively.

1. [↑](#footnote-ref-1)
2. [↑](#footnote-ref-2)
3. [↑](#footnote-ref-3)
4. [↑](#footnote-ref-4)
